# CCL4 as a potential serum factor in differential diagnosis of central nervous system inflammatory diseases and gliomas

**DOI:** 10.3389/fimmu.2024.1461450

**Published:** 2024-09-19

**Authors:** Tian-Jie Lyu, Jia Wang, Fengmao Zhao, Ke Sun, Zheng Zhao, Runfa Tian, Zhendong Guo, Haoran Wang, Xin Zhao, Wenping Ma, Mingshan Zhang, Wangshu Xu

**Affiliations:** ^1^ Department of Neurology, Beijing Tiantan Hospital, Capital Medical University, Beijing, China; ^2^ Department of Neurology, China National Clinical Research Center for Neurological Diseases, Beijing, China; ^3^ Department of Neurosurgery, Beijing Children’s Hospital, Capital Medical University, National Center for Children’s Health, Beijing, China; ^4^ Functional Neurosurgery Department, National Center for Children’s Health, Beijing Children’s Hospital, Capital Medical University, Beijing, China; ^5^ Beijing Neurosurgical Institute, Capital Medical University, Beijing, China; ^6^ Department of Neurosurgery, Beijing Tiantan Hospital, Capital Medical University, Beijing, China; ^7^ Department of Pulmonary and Critical Care Medicine, Cangzhou People’s Hospital, Cangzhou, China; ^8^ Department of Neurosurgery, Sanbo Brain Hospital, Capital Medical University, Beijing, China; ^9^ Innovation Center, Beijing Children’s Hospital, Capital Medical University, National Center for Children’s Health, Beijing, China

**Keywords:** gliomas, central nervous system inflammatory diseases, non-invasive testing, non-invasive testing of biological structures, potential biomarker, clinical diagnosis

## Abstract

Computed tomography (CT) scans and magnetic resonance imaging (MRI) are commonly utilized to detect brain gliomas and central nervous system inflammation diseases. However, there are instances where depending solely on medical imaging for a precise diagnosis may result in unsuitable medications or treatments. Pathological analysis is regarded as the definitive method for diagnosing brain gliomas or central nervous system inflammation diseases. To achieve this, a craniotomy or stereotaxic biopsy is necessary to collect brain tissue, which can lead to complications such as cerebral hemorrhage, neurological deficits, cerebrospinal fluid leaks, and cerebral edema. Consequently, the advancement of non-invasive or minimally invasive diagnostic techniques is currently a high priority. This study included samples from four glioma patients and five patients with central nervous system inflammatory diseases, comprising both serum and paired cerebrospinal fluid (CSF). A total of 40 human cytokines were identified in these samples. We utilized a receiver operating characteristic (ROC) analysis to assess the sensitivity and specificity for distinguishing central nervous system inflammation diseases and gliomas. Additionally, we examined the correlation of these factors between serum and CSF in the patients. Ultimately, the identified factors were validated using serum from patients with clinically confirmed gliomas and central nervous system inflammation diseases followed by detection and statistical analysis through ELISA. The levels of serum factors IL-4, IFN-α, IFN-γ, IL-6, TNF-α, CCL4, CCL11, and VEGF were found to be significantly higher in gliomas compared with inflammatory diseases of the central nervous system (p < 0.05). Furthermore, a strong correlation was observed between the levels of CCL4 in serum and CSF, with a correlation coefficient of r = 0.92 (95% CI = 0.20–0.99, p = 0.027). We gathered more clinical samples to provide further validation of the abundance of CCL4 expression. A clinical study analyzing serum samples from 19 glioma patients and 22 patients with central nervous system inflammation diseases revealed that CCL4 levels were notably elevated in the inflammatory group compared with the glioma group (p < 0.001). These results suggest that assessing serum CCL4 levels may be useful in distinguishing those patients for clinical diagnostic purposes.

## 1 Introductions

Brain tumors are among the deadliest types of tumors and contribute significantly to illness and death globally (1). It is estimated that brain tumors occur at a rate of 10 cases per 100,000 individuals annually (2). Gliomas are the most prevalent form of malignant brain tumor, making up 81% of all brain tumors, with an incidence rate of seven cases per 100,000 people (3). Adult brain tumors have a high mortality rate, particularly gliomas (4). In contrast, intracranial demyelinating inflammation has a lower mortality rate, but their imaging characteristics can be easily mistaken for one another, making it challenging to rely solely on medical imaging for accurate identification. Gliomas and central nervous system inflammatory diseases can be differentiated based on their underlying difference mechanisms, treatment approaches, and prognoses (5). Gliomas are typically managed through a combination of methods, including surgical removal, followed by chemotherapy and radiotherapy, as well as biological therapies like tumor treating fields (TTF) (6–9). Most central nervous system inflammatory diseases are multiple sclerosis, neuromyelitis optica, concentric sclerosis, disseminated encephalomyelitis, and others, which can lead to the formation of tumor-like demyelinating lesions (10–12). Immune therapy, hormone therapy, CD20 therapy to eliminate antibodies, or plasma exchange are necessary treatments (13–15). Brain tumors can develop at any age, but they are more frequently seen in young adults, middle-aged individuals, and older adults. They are present in 2% of standard autopsies. Therefore, there is an urgent need to discover novel biomarker that are sensitive, specific, and easy to practice (16).

Given the challenging circumstances surrounding imaging diagnosis and treatment, surgical biopsy is primarily used to collect tissue samples from lesions, allowing for pathological diagnosis to guide treatment. However, this procedure may lead to bleeding or nerve damage. Therefore, there is an urgent need to identify less invasive or non-invasive detection methods that can accurately differentiate between brain inflammations and gliomas.

In our study, we examined 40 abundance factors in CSF and serum related to gliomas and central nervous system inflammatory diseases. We assessed the correlation of these immune factors and compared them between the glioma group and the central nervous system inflammatory diseases group in both CSF and serum. We found that CCL4 levels were notably elevated in cases of encephalitis compared with glioma, suggesting that it could serve as a potential biological marker.

## 2 Materials and methods

### 2.1 Patients’ information

In our study, we analyzed samples from nine patients, comprising four with gliomas and five with central nervous system inflammatory diseases, to assess 40 human cytokines. In addition, we included CSF and serum from eight patients with cranial hypertension as a control group to validate the previous results. The diagnoses of the four glioma patients were confirmed through imaging and pathology. The five patients with central nervous system inflammatory diseases were diagnosed with myelitis (two patients), inflammatory demyelinating disease of the central nervous system (two patients), and tumor-like demyelinating lesions. Serum tests and C-reactive protein levels in these five patients indicated abnormal findings, but they showed improvement in clinical symptoms and imaging lesions following anti-inflammatory treatment.

### 2.2 Analysis flowchart of 40 human cytokines

Human cytokine analysis was conducted using the ABplex Human Cytokine 40-Plex Assay Kit (Cat. No: RK04368), supplied by Beijing Qiangxin Biorepublic Co., Ltd., in Beijing, China. The ABplex Human Cytokine 40-Plex Assay Kit is designed for the simultaneous quantification of 40 different human cytokines, chemokines, and growth factors in various sample types, including serum, plasma, and cell culture supernatants.

It allows simultaneous measurement of 40 analytes in a single well, increasing throughput and conserving sample volume. This kit is commonly used in immunology, oncology, and infectious disease research to analyze cytokine responses and to identify and validate biomarkers in various disease states (17).

After collecting samples from patients, including serum and CSF from various groups, the standards and reagents were prepared. Then, 50 μL of standards or test samples was added to each well, followed by 50 μL of coded microspheres and 50 μL of the working detection antibody. The mixture was incubated for 30 min at 37°C and washed once, and then 50 μL of SA-PE was added. This was incubated for 15 min at 37°C in the dark, followed by the addition of 70 μL of wash buffer. Detection was performed using the ABplex-100.

### 2.3 Patients’ inclusion criteria for ELISA

Patients with gliomas were diagnosed using a combination of pathological and imaging techniques, with evaluations conducted by two different neuropathologists and two independent imaging experts. Serum samples were obtained from 19 glioma patients prior to surgery at Tiantan Hospital. Furthermore, 22 patients with inflammation were identified through imaging and serum C-reactive protein tests, and their serum was also collected at Tiantan Hospital.

### 2.4 ELISA flowchart

At 4°C overnight, 100 ng of recombinant CCL4 in carbonate buffer (pH 9.6) was added to 96-well ELISA plates (MM-61999H1, No. 2024022699H). After a 2-h blocking period at room temperature with 300 µL of blocking buffer (10 mM pH 7.4 PBS with 15% goat serum), serum samples were added to the wells and incubated for 1 h at 37°C, followed by five washing steps. Then, 100 µL of diluted HRP-labeled goat anti-human IgG antibody (Jiangsu Meimian Industrial Co., Ltd., China) was added and incubated for another hour at 37°C. After three washes with the washing buffer, the plates were treated with 50 µL of 2 mol/L H_2_SO_4_ and 100 µL of TBM per well for 15 min at 37°C to stop the reaction. A microplate reader set to 450 nm was used to measure the optical density of each well over a period of 5 min.

### 2.5 Specimen collection

The matched serum and CSF were enrolled for analysis. Those samples were collected from Beijing Tiantan Hospital and Beijing Children’s Hospital. All patients were diagnosed with brain tumors or different kinds of brain inflammation. These glioma patients were diagnosed by two independent neuropathologists according to the 2021 WHO classification. The brain inflammation was diagnosed by two independent clinical neurologists and ancillary examination. In addition, weighted kappa analysis was used to evaluate the consistency of the gliomas and brain inflammation.

### 2.6 Statistical analysis

Data analysis and graphing were conducted using GraphPad Prism version 9.0.0 (San Diego, CA, USA). To assess concentration differences between brain inflammation and brain tumors, we utilized two-way ANOVA along with Sidak’s *post-hoc* test to adjust for multiple comparisons of serum and CSF from the same patient. We also carried out ROC curve analysis to evaluate whether any of the measured immunological molecules could distinguish between brain inflammation and brain tumors. This analysis yielded information on cutoff values, area under the curve (AUC) values, and specificities, sensitivities, and p values. Cutoff values were determined through ROC analyses by optimizing the Youden index (sensitivity + specificity − 1) and selecting the most favorable likelihood ratio. Lastly, we applied the Pearson correlation coefficient to assess the relationships between the concentrations of immunological molecules in serum and CSF. Statistically significant differences were considered those with a p value of less than 0.05.

## 3 Results

**Figure 1:**
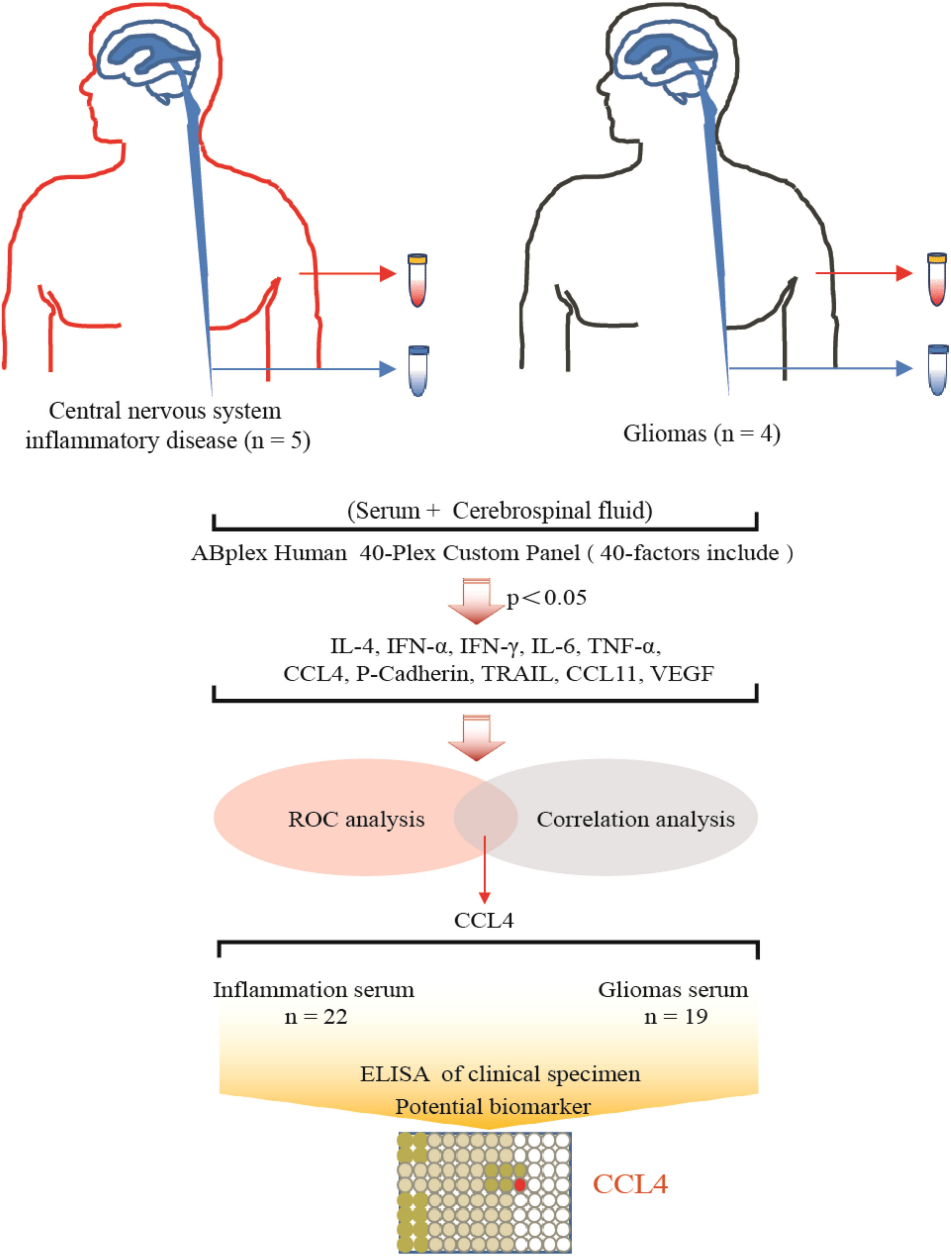
Summary graphical chart for this research. Brain inflammation groups (n=5) and brain glioma groups (n=4) samples were included in the research. Totally 40 immunological factors were analyzed. Two-way ANOVA, ROC analysis, and Pearson correlation coefficient analysis were performed, with the p value <0.05. ELISA of patients’ serum was analyzed.

**Figure 2:**
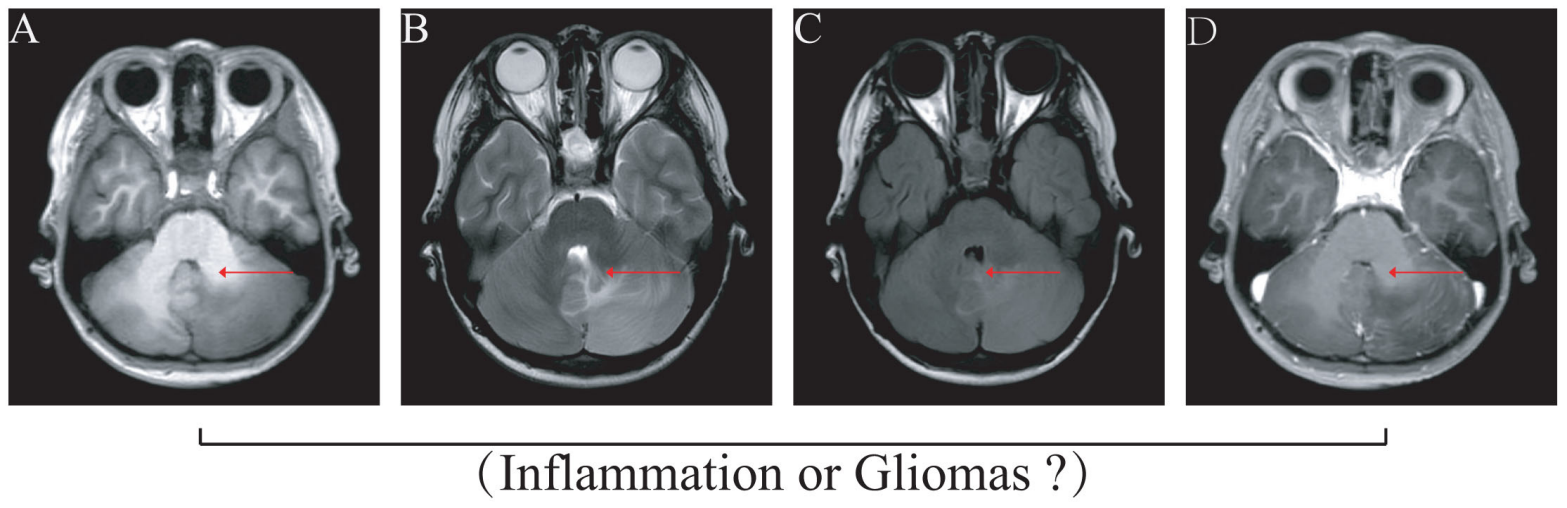
Imaging illustrates that differentiating glioma from inflammation disease is difficult. The images of MRI difficulty to gliomas or intracranial demyelinating inflammation disease. **(A)** T1 image, **(B)** T2 image, **(C)** FLAIR (fluid-attenuated inversion recovery), **(D)** Enhanced nuclear magnetism.

**Figure 3:**
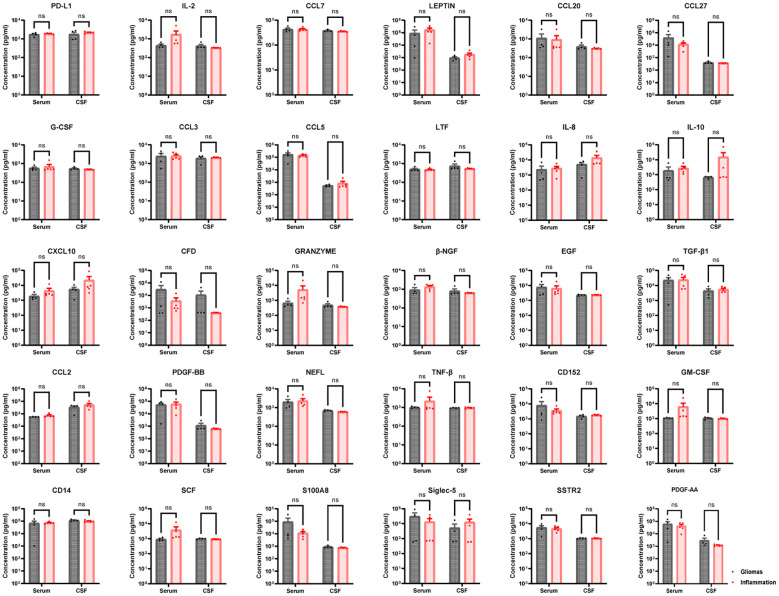
Thirty no-sense factors compared in gliomas and inflammation group. PD-L1, IL-2, CCL7, LEPTIN, CCL20, CCL27, G-CSF, CCL3, CCL5, LTF, IL-8, IL-10, CCL10, CFD, GRANZYME, β-NGF, EGF, TGF-β1, CCL2, PDGF-BB, NEFL, TNF-β, CD152, GM-CSF, CD14, SCF, S100A8, Siglec-5, SSTR2, and S100A8—30 factors in serum had non-sense in brain inflammation groups than brain gliomas groups (p > 0.05). ns, not significant.

**Figure 4:**
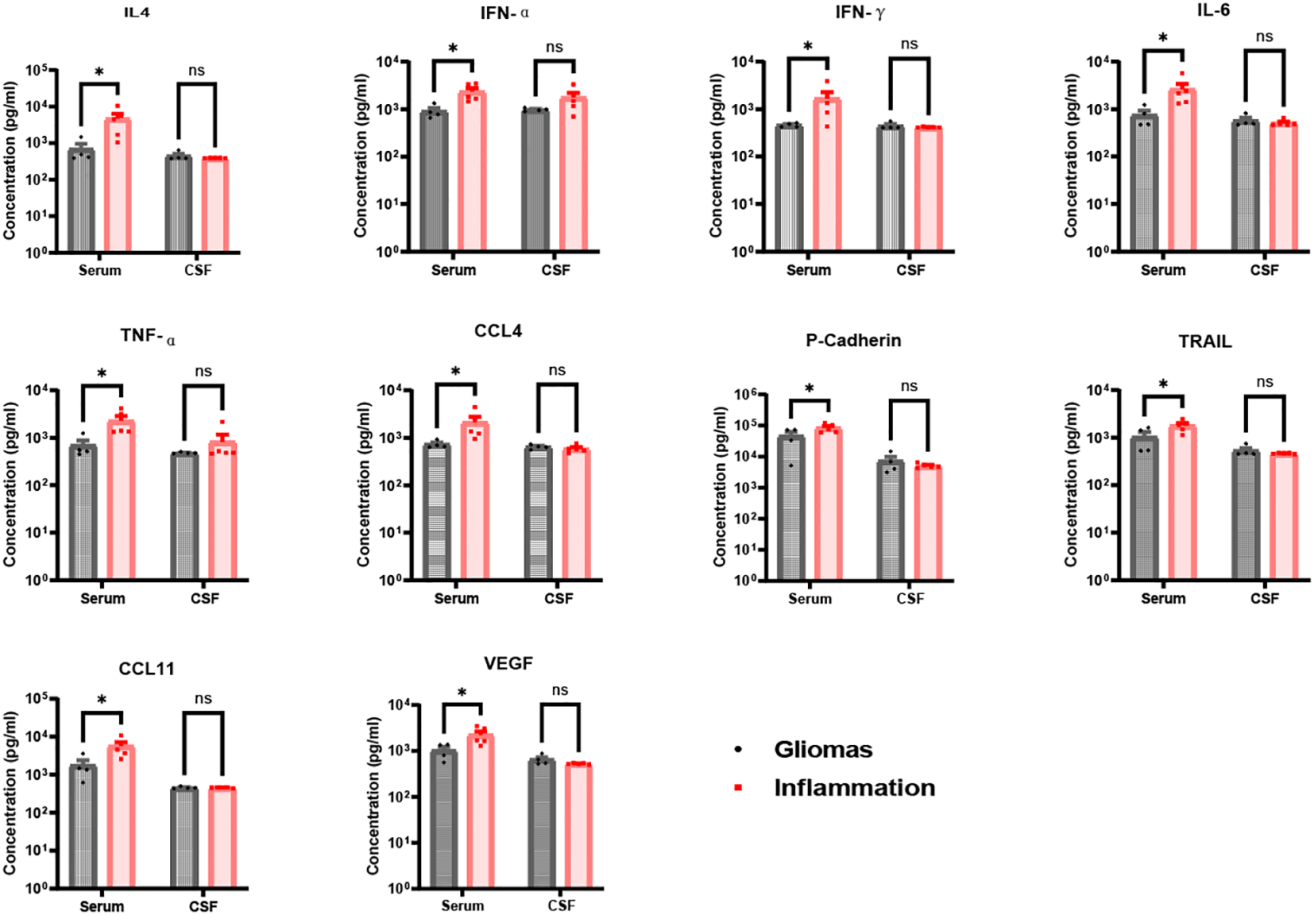
Ten significance factors compared in brain gliomas and inflammation group. IL-4, IFN-α, IFN-γ, IL-6, TNF-α, CCL4, P-Cadherin, TRAIL, CCL11, and VEGF factors in serum were significantly in brain inflammation groups than brain gliomas groups (IL-4 p = 0.023, IFN-α p = 0.027, IFN-γ p = 0.049, IL-6 p = 0.028, TNF-α p = 0.024, CCL4 p = 0.038, P-Cadherin p = 0.035, TRAIL p = 0.022, CCL11 p = 0.013, VEGF p = 0.012), but less significance in CSF between brain inflammation than brain tumor (p > 0.05). * p <0.05; ns, not significant.

A total of four gliomas and five inflammatory diseases of the central nervous system were examined. An analysis of 40 human cytokines was performed using the ABplex Human Cytokine 40-Plex Assay Kit on serum or CSF samples via a non-invasive method (**Figure 1**). The weighted kappa values for the glioma patients as determined by two neuropathologists according to the 2021 WHO classification was 0.81, and the weighted kappa values for the brain inflammation as determined by two independent clinical neurologists was 0.73. There are cases where it is challenging to distinguish between intracranial inflammation and glioma using imaging methods like T1, T2, FLAIR, and T1 enhancement (**Figure 2**). Our study found that 30 factors exhibited no notable differences in serum and cerebrospinal fluid (**Figure 3**). Pathological analysis is essential for identifying the specific type of disease. However, procedures like craniotomy or biopsy may risk normal neurological function. Thus, there is a pressing need for non-invasive or minimally invasive methods to improve diagnostic accuracy for these conditions. Our research revealed that over 30 factors showed no significant differences in serum and CSF (**Figure 4**). On the basis of these results, we used CSF and serum from patients with intracranial hypertension for control and showed that CCL4 was significantly higher in both the inflammatory and tumor groups than in the control group (**Supplementary Figures 1**, **2**).

**Figure 5:**
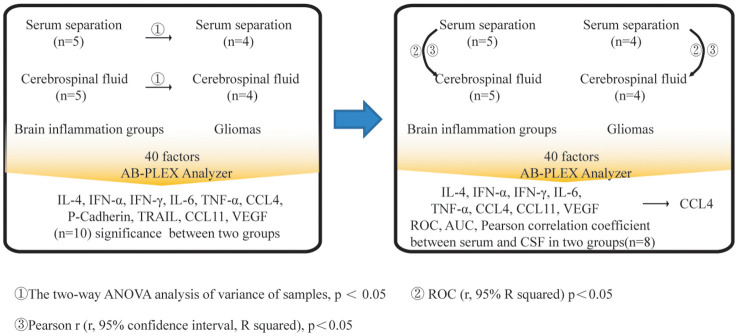
Process for screening and analysis of CCL4. Firstly, 40 immunological factors were detected in serum or CSF between brain inflammation and brain gliomas groups. The two-way ANOVA analysis of variance of samples was evaluated, p < 0.05. Secondly, ROC (r, 95% R squared) was evaluated, p < 0.05. Thirdly, Pearson r (r, 95% confidence interval, R squared) was evaluated, p<0.05. CCL4 had the significant difference among those comparisons, p < 0.05.

**Figure 6:**
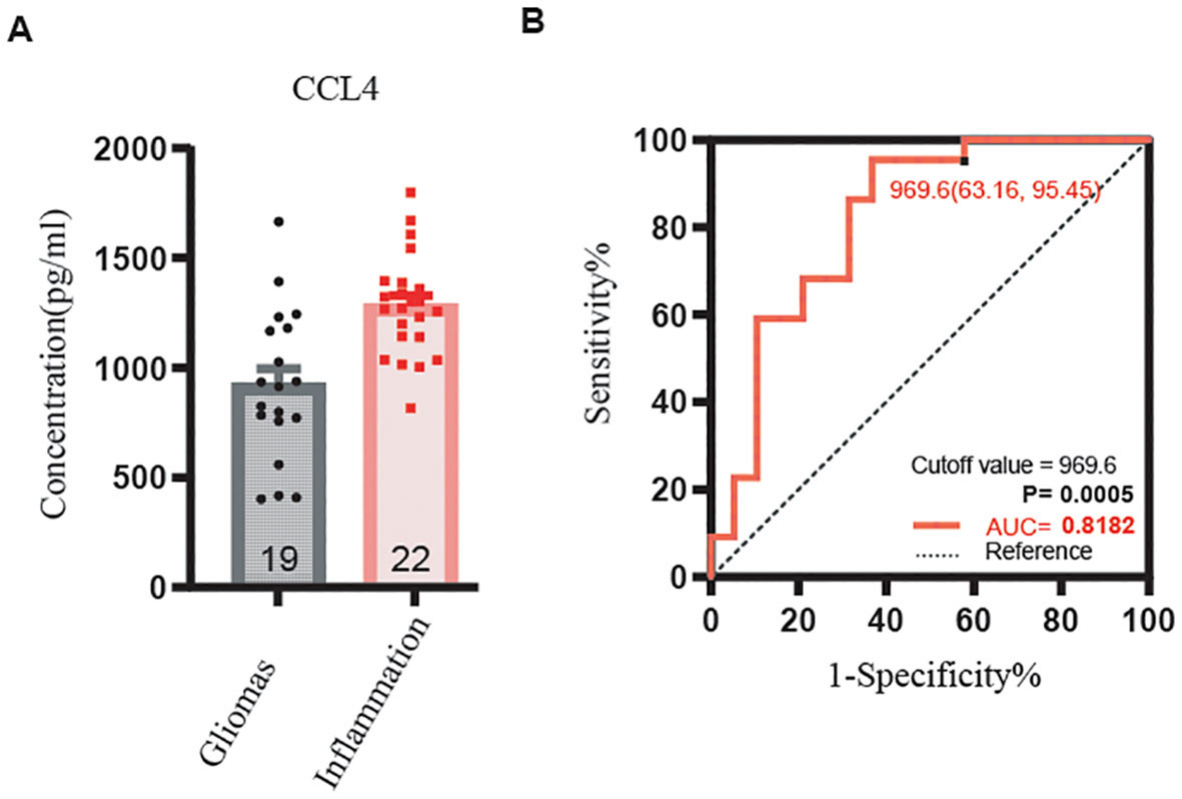
CCL4 serum expressing level in gliomas and inflammation by ELISA and ROC curve of CCL4. **(A)** The expression level of CCL4 was lower in gliomas than inflammation, p < 0.001. **(B)** The receiver operating characteristic (ROC) curve for CCL4. Sensitivity is listed on the y-axis, and 1-specificity is listed on the x-axis.

In contrast, levels of IL-4, IFN-α, IFN-γ, IL-6, TNF-α, CCL4, P-cadherin, TRAIL, CCL11, and VEGF were significantly higher in serum than in CSF for both glioma and brain inflammation groups. However, there were no differences in CSF levels between the glioma and brain inflammation groups. The specific p-values for these factors were as follows: IL-4 p = 0.023, IFN-α p = 0.027, IFN-γ p = 0.049, IL-6 p = 0.028, TNF-α p = 0.024, CCL4 p = 0.038, P-cadherin p = 0.035, TRAIL p = 0.022, CCL11 p = 0.013, and VEGF p = 0.012 (**Figure 4**). ROC analysis of immunological molecules in serum and CSF showed that the expression levels of IL-4, IFN-α, IFN-γ, IL-6, TNF-α, CCL4, CCL11, and VEGF exhibited high sensitivity and specificity in distinguishing between brain inflammation and brain tumor groups (p < 0.05) (**Table 1**). In our correlation analysis in serum and CSF, we found that CCL4 was significantly correlated between serum and CSF in the brain inflammation group (r = 0.92, 95% CI = 0.20–0.99, p = 0.027) (**Figure 5**, **Table 2**), whereas no other factors showed significant differences between groups. To further assess the effectiveness of CCL4, the researchers gathered more data from the serum of 19 patients diagnosed with glioma and 22 patients suffering from brain inflammation. The results showed a notable rise in CCL4 levels, indicating that elevated serum CCL4 expression could be a marker for intracranial inflammatory diseases (**Figure 6**). ROC analysis showed a cutoff value of 969.6 and an AUC of 0.8182.

**Table 1:** ROC analysis of immunological molecules in CSF and serum.

Cytokines	Sample	Cutoff (pg/ml)	Sensitivity (95% CI) [%]	Specificity (95% CI) [%]	AUCs (95% CI)	P-value
IL-4	Serum	> 1593	80.00 (37.55-98.97)	100.0 (51.01-100)	0.95 (0.81-1.00)	* 0.0275
	CSF	> 385.5	100.0 (56.55-100)	50.0 (8.89-91.12)	0.50 (0.01-0.99)	>0.9999
IFN-α	Serum	> 1402	100.0 (56.55-100)	100.0 (51.01-100)	1.00 (1.00-1.00)	* 0.0143
	CSF	> 1119	80.00 (37.55-98.97)	100.0 (51.01-100)	0.80 (0.45-1.00)	0.1416
IFN-γ	Serum	> 681.0	80.00 (37.55-98.97)	100.0 (51.01-100)	0.90 (0.68-1.00)	* 0.0500
	CSF	< 412.0	80.00 (37.55-98.97)	50.00 (8.89-81.12)	0.70 (0.34-1.00)	0.3272
IL-6	Serum	> 1275	100.0 (56.55-100)	100.0 (51.01-100)	1.00 (1.00-1.00)	* 0.0143
	CSF	< 491.5	80.00 (37.55-98.97)	75 (30.06-98.72)	0.75 (0.41-1.00)	0.2207
TNF-α	Serum	> 1288	100.0 (56.55-100)	100.0 (51.01-100)	1.00 (1.00-1.00)	* 0.0143
	CSF	> 476.0	80.00 (37.55-98.97)	75 (30.06-98.72)	0.75 (0.41-1.00)	0.2207
CCL4	Serum	> 936.0	100.0 (56.55-100)	100.0 (51.01-100)	1.00 (1.00-1.00)	* 0.0143
	CSF	< 611.0	80.00 (37.55-98.97)	75 (30.06-98.72)	0.70 (0.32-1.00)	0.3272
P-Cadherin	Serum	> 75108	60 (23.07-92.89)	100.0 (51.01-100)	0.80 (0.49-1.00)	0.1416
	CSF	> 4176	100.0 (51.01-100)	50.0 (8.89-91.12)	0.50 (0.01-0.99)	>0.9999
TRAIL	Serum	> 1748	60 (23.07-92.89)	100.0 (51.01-100)	0.85 (0.59-1.00)	0.0864
	CSF	> 459.5	80.00 (37.55-98.97)	50.0 (8.89-91.12)	0.50 (0.08-0.92)	>0.9999
CCL11	Serum	> 3645	80.00 (37.55-98.97)	100.0 (51.01-100)	0.95 (0.81-1.00)	* 0.0275
	CSF	> 452.5	80.00 (37.55-98.97)	75 (30.06-98.72)	0.65 (0.23-1.00)	0.4624
VEGF	Serum	> 1511	80.00 (37.55-98.97)	100.0 (51.01-100)	0.90 (0.68-1.00)	* 0.0500
	CSF	< 522.0	60 (23.07-92.89)	100.0 (51.01-100)	0.80 (0.49- 1.00)	0.1416

* p <0.05.

Bold values indicates p-values < 0.05.

**Table 2:** Correlation analysis of immunological molecules in CSF and serum.

Cytokines	Patient group	Pearson r (95% CI)	R squared	P value (two-tailed)
IL-4	Tumor	−0.20 (−0.97 - 0.94)	0.0403	0.7992
	Inflammation	−0.55 (−0.96 - 0.64)	0.3047	0.3347
IFN-α	Tumor	0.76 (-0.75 - 0.99)	0.5719	0.2438
	Inflammation	0.15 (−0.84 - 0.91)	0.0234	0.8062
IFN-γ	Tumor	0.43 (−0.91 - 0.98)	0.1808	0.5747
	Inflammation	−0.39 (−0.95 - 0.75)	0.1535	0.5143
IL-6	Tumor	0.17 (−0.95 - 0.97)	0.0275	0.8342
	Inflammation	−0.29 (−0.93 - 0.80)	0.0844	0.6354
TNF-α	Tumor	−0.11 (−0.97 - 0.95)	0.0118	0.8915
	Inflammation	−0.39 (−0.95 - 0.75)	0.1554	0.5114
CCL4	Tumor	−0.69 (−0.99 - 0.81)	0.4730	0.3122
	Inflammation	0.92 (0.20 - 0.99)	0.8456	* 0.0270
P-Cadherin	Tumor	0.71 (−0.79 - 0.99)	0.5050	0.2894
	Inflammation	0.34 (−0.92 - 0.98)	0.1139	0.6626
TRAIL	Tumor	0.43 (−0.90 - 0.98)	0.1888	0.5655
	Inflammation	0.08 (−0.86 - 0.90)	0.0063	0.8992
CCL11	Tumor	−0.45 (−0.98 - 0.90)	0.1996	0.5533
	Inflammation	0.45 (−0.72 - 0.95)	0.1987	0.4518
VEGF	Tumor	−0.58 (−0.99 - 0.86)	0.3323	0.4236
	Inflammation	0.23 (−0.82 - 0.92)	0.0517	0.7131

Bold values indicates p-values < 0.05.

## 4 Discussion

To detect gliomas or intracranial inflammatory diseases, gadolinium-enhanced T1-weighted MRI or contrast-enhanced CT scans are utilized. Imaging tests can occasionally complicate the distinction between the two disease types, necessitating a biopsy for a clearer understanding of the disease’s pathology (18, 19). There is an urgent need for non-invasive techniques to distinguish between intracranial tumors and inflammation (20, 21).

In our study, we gathered samples from four brain glioma diagnoses along with corresponding pathological diagnoses, serum, and CSF. We also collected serum samples from five cases of brain inflammation, matched with CSF. Using these serum samples, we aimed to identify potential factors that could differentiate between brain gliomas and brain inflammation. Our findings revealed that the serum levels of IL-4, IFN-α, IFN-γ, IL-6, TNF-α, CCL4, P-cadherin, TRAIL, CCL11, and VEGF were significantly higher in cases of brain inflammation compared with brain gliomas. However, we did not find any significant factors in the CSF samples. Additionally, we examined the relationships between serum and CSF in both brain glioma and brain inflammation groups. Notably, we discovered a strong correlation between the serum and CSF levels of CCL4 in the brain inflammation group. To confirm our findings, we collected serum again from clinical patients diagnosed with brain inflammation disease and glioma and measured the CCL4 levels present. Our results indicated that the expression of CCL4 in cases of brain inflammation disease was notably higher compared with that in glioma, and this difference was statistically significant (p < 0.001, AUC = 0.8182). CCL4 may serve as a potential biomarker to distinguish between brain gliomas and brain inflammation in serum.

CCL4, or macrophage inflammatory protein-1 beta (MIP-1β), is a chemokine that plays a significant role in the immune response. It serves as a crucial chemokine that attracts and activates immune cells, influences the immune response, and encourages inflammation. When it binds to the CCR5 receptor, it activates several signaling pathways, such as NF-κB, MAPK, and JAK/STAT, all of which play a role in disease development. In the context of gliomas, CCL4 aids in tumor progression by altering the tumor microenvironment, thereby facilitating tumor growth, invasion, and the formation of new blood vessels. In low-grade gliomas (LGG), CCL4 is a key element for the survival of LGG stem cells (22). Studies have shown that CCL4 can enhance tumor growth by attracting factors that promote tumorigenesis, and it also interacts with fibroblasts and endothelial cells to create a tumor microenvironment that supports tumor development (23, 24). Additionally, CCL4 facilitates the migration of osteosarcoma cells by activating signaling pathways such as focal adhesion kinase (FAK), protein kinase B (AKT), and hypoxia-inducible factor 1 subunit alpha (HIF-1α). Our goal is to discover a minimally invasive or non-invasive approach to identify brain gliomas and brain inflammation. To achieve this, we collected serum and CSF samples from four brain glioma patients and five with brain inflammation for immune factor analysis and statistical evaluation. We also measured CCL4 levels in confirmed clinical serum samples using ELISA. Our findings indicate that serum CCL4 could serve as a potential marker for identifying between intracranial inflammation disease and gliomas.

This study offers important insights into the potential of serum CCL4 levels as a biomarker for differentiating between brain gliomas and central nervous system inflammatory diseases, but it also has several limitations. Firstly, the sample size was relatively small, especially in the initial analysis, which may restrict the applicability of the results. To validate the diagnostic value of CCL4 in various populations, larger and more diverse groups are needed. Secondly, the research concentrated on a specific group of cytokines, potentially overlooking other relevant biomarkers that could be significant. Future investigations should include a wider range of biomarkers to create a more thorough diagnostic tool. Additionally, it is clinically difficult to obtain cerebrospinal fluid and serum from completely normal individuals; therefore, cerebrospinal fluid and serum from patients with intracranial hypertension were included in this study as a control group. Moreover, although a strong correlation between serum and cerebrospinal fluid CCL4 levels was found, the study did not investigate the mechanisms behind this relationship, which is essential for understanding the pathophysiology of these diseases. Lastly, the research did not consider possible confounding factors such as patient comorbidities, medication use, or other neurological conditions that might affect cytokine levels and the accuracy of the biomarker. Future studies should aim to confirm its clinical relevance, explore its mechanistic functions, and develop targeted therapies. These initiatives will contribute to personalized medicine strategies, ultimately enhancing patient care and outcomes.

## 5 Conclusion

This research reveals a significant relationship between CCL4 concentrations in serum and cerebrospinal fluid, indicating its possible role as a biomarker. Additional validation indicated that serum CCL4 levels were notably higher in patients with central nervous system inflammation than in those with gliomas. These results imply that serum CCL4 levels may act as a non-invasive biomarker to differentiate between gliomas and inflammatory conditions, providing a valuable resource for enhancing diagnostic precision and potentially minimizing the necessity for invasive diagnostic methods in clinical settings.

## Data Availability

The original contributions presented in the study are included in the article/**Supplementary Material**. Further inquiries can be directed to the corresponding authors.

## Supplementary material

Supplementary Figure 1 Comparison of CCL4 expression abundance between intracranial hypertension control group and glioma group. (p < 0.05).

Supplementary Figure 2 Comparison of CCL4 expression abundance between intracranial hypertension control group and inflammation group. (p < 0.05).
