# Supplementary figures and images for: CCL4 as a potential serum factor in differential diagnosis of central nervous system inflammatory diseases and gliomas
Source: Front Immunol. 2024 Sep 19;15:1461450. doi: 10.3389/fimmu.2024.1461450 (PMC11446780; doi:10.3389/fimmu.2024.1461450)

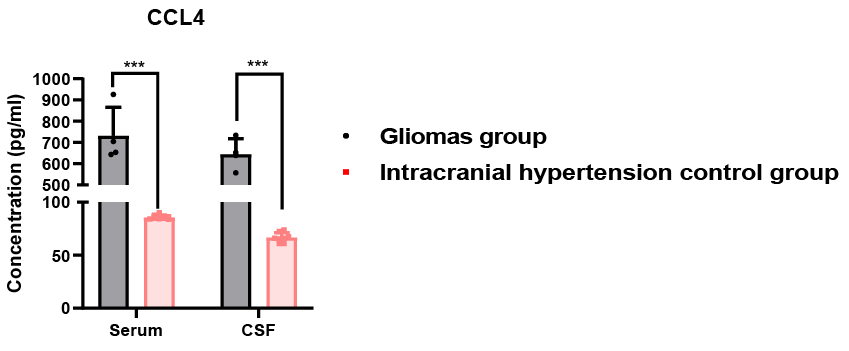

Supplement: Supplementary Figure 1 — Comparison of CCL4 expression abundance between intracranial hypertension control group and glioma group. (p < 0.05). [file Image1.tif]

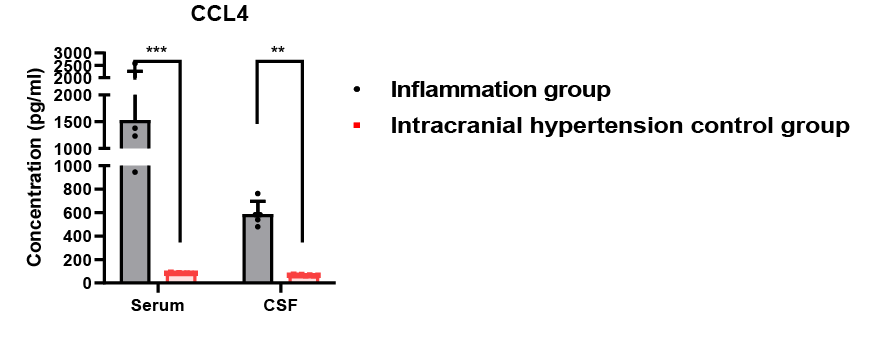

Supplement: Supplementary Figure 2 — Comparison of CCL4 expression abundance between intracranial hypertension control group and inflammation group. (p < 0.05). [file Image2.tif]
