# Supplementary material for: CCL4 as a potential serum factor in differential diagnosis of central nervous system inflammatory diseases and gliomas
Source: Front Immunol. 2024 Sep 19;15:1461450. doi: 10.3389/fimmu.2024.1461450 (PMC11446780; doi:10.3389/fimmu.2024.1461450)
Supplement: Supplementary file 3 [file DataSheet1.doc]

| Supprementary Table 1. Intracranial hypertension contral and disease groups | | | | | |  |
| --- | --- | --- | --- | --- | --- | --- |
| Group | n | Sex | Age(Year) | Serum CCL4 level (pg/ml) | CSFC CL4 level (pg/ml) | Disease type |
| Inflammation group | Patient 1 | F | 58 | 480 | 1231 | Myelitis |
|  | Patient 2 | M | 43 | 763 | 4464 | Inflammatory demyelinating disease |
|  | Patient 3 | F | 39 | 583 | 2581 | Inflammatory demyelinating disease |
|  | Patient 4 | F | 11 | 540 | 945 | Demyelinating disease |
|  | Patient 5 | F | 70 | 583 | 1377 | Myelitis |
|  |  |  |  |  |  |  |
| Gliomas group | Patient 1 | M | 63 | 557 | 927 | WHO 4 grade |
|  | Patient 2 | M | 55 | 653 | 644 | WHO 4 grade |
|  | Patient 3 | M | 37 | 639 | 654 | WHO 4 grade |
|  | Patient 4 | M | 52 | 734 | 705 | WHO 4 grade |
|  |  |  |  |  |  |  |
| Control group | Patient 1 | F | 48 | 65.96838241 | 84.12339735 | Intracranial hypertension |
|  | Patient 2 | M | 29 | 72.27244448 | 84.75399148 | Intracranial hypertension |
|  | Patient 3 | F | 29 | 61.13547831 | 83.91492249 | Intracranial hypertension |
|  | Patient 4 | M | 33 | 61.13714254 | 87.71531107 | Intracranial hypertension |
|  | Patient 5 | F | 33 | 73.8312499 | 84.39005717 | Intracranial hypertension |
|  | Patient 6 | M | 42 | 67.51385219 | 86.1983203 | Intracranial hypertension |
|  | Patient 7 | F | 47 | 66.11142677 | 89.96777248 | Intracranial hypertension |
|  | Patient 8 | M | 35 | 65.39062775 | 84.83119279 | Intracranial hypertension |
