# Supplementary material for: CCL4 as a potential serum factor in differential diagnosis of central nervous system inflammatory diseases and gliomas
Source: Front Immunol. 2024 Sep 19;15:1461450. doi: 10.3389/fimmu.2024.1461450 (PMC11446780; doi:10.3389/fimmu.2024.1461450)
Supplement: Supplementary file 4 [file DataSheet2.doc]

| Supplementary Figure 2. Patients' clinical Information for ELISA | | | |
| --- | --- | --- | --- |
|  | | | |
|  | Gliomas | Inflammation | p value |
| Age(Year) | 51.26±15.28 | 52.95±15.41 | ＞0.05 |
| Sex(M/F) | 10/9 (n=19) | 10/12 (n=22) | ＞0.05 |
| Serum CCL4 level (pg/ml) | 18.33±17.66 | 25.58±4.71 | ＜0.01 |
