# Supplementary material for: CCL4 as a potential serum factor in differential diagnosis of central nervous system inflammatory diseases and gliomas
Source: Front Immunol. 2024 Sep 19;15:1461450. doi: 10.3389/fimmu.2024.1461450 (PMC11446780; doi:10.3389/fimmu.2024.1461450)
Supplement: Supplementary file 5 [file DataSheet3.doc]

| Supplementary Table 3 Abbreviation | | | |  |
| --- | --- | --- | --- | --- |
|  |  |  |  | |
| CT: Computed Tomography |  |  |  | |
| MRI: Magnetic Resonance Imaging |  |  |  | |
| FLAIR: Fluid attenuated inversion recovery |  |  |  | |
| CSF: Cerebrospinal fluid |  |  |  | |
| ROC: Receiver operating characteristic |  |  |  | |
| LGG: Low-grade gliomas |  |  |  | |
| FAK: Focal adhesion kinase |  |  |  | |
| HIF-1α：Hypoxia-inducible factor 1 subunit alpha |  |  |  | |
| MIP-1β: macrophage inflammatory protein-1 beta |  |  |  | |
